# Adaptive Sampling Framework for Imbalanced DDoS Traffic Classification

**DOI:** 10.3390/s25133932

**Published:** 2025-06-24

**Authors:** Hongjoong Kim, Deokhyeon Ham, Kyoung-Sook Moon

**Affiliations:** 1 Department of Mathematics, Korea University, 145 Anam-ro, Seongbuk-gu, Seoul 02841, Republic of Korea; hongjoong@korea.ac.kr (H.K.); hamch6910@korea.ac.kr (D.H.); 2 Department of Finance and Big Data, Gachon University, 1342 Seongnamdaero, Sujeong-gu, Seongnam-si 13120, Gyeonggi-do, Republic of Korea

**Keywords:** imbalanced dataset, DDoS, classification, sampling methods

## Abstract

Imbalanced data is a major challenge in network security applications, particularly in DDoS (Distributed Denial of Service) traffic classification, where detecting minority classes is critical for timely and cost-effective defense. Existing machine learning and deep learning models often fail to accurately classify such underrepresented attack types, leading to significant degradation in performance. In this study, we propose an adaptive sampling strategy that combines oversampling and undersampling techniques to address the class imbalance problem at the data level. We evaluated our approach using benchmark DDoS traffic datasets, where it demonstrated improved classification performance across key metrics, including accuracy, recall, and F1-score, compared to baseline models and conventional sampling methods. The results indicate that the proposed adaptive sampling approach improved minority class detection performance under the tested conditions, thereby improving the reliability of sensor-driven security systems. This work contributes a robust and adaptable method for imbalanced data classification, with potential applications across simulated sensor environments where anomaly detection is essential.

## 1. Introduction

In the era of the Internet of Things (IoT), the proliferation of sensor-driven data streams has introduced new opportunities and challenges in the domain of cybersecurity. Recent studies have highlighted the growing range of cybersecurity threats faced by sensor-based systems, including IoT networks and autonomous platforms. For example, [1] emphasizes the dangers of real-time sensor attacks in autonomous systems, while [2] reports that IoT infrastructures are particularly vulnerable to DDoS and data manipulation attacks due to their limited processing capacity and weak security protocols. These threats underscore the critical need for effective early detection mechanisms, particularly in scenarios involving imbalanced data, where critical anomalies are infrequent.

Among these, Distributed Denial of Service (DDoS) attacks pose a particularly critical threat. For instance, Ref. [3] analyzes the structure of DDoS attacks and highlights their rapidly evolving tactics, while [4] presents a taxonomy of DDoS detection methods, emphasizing the difficulty in early-stage detection. To counter these threats, machine learning and deep learning approaches have been increasingly adopted. Ref. [5] introduces a convolutional neural network-based method for detecting abnormal traffic patterns in real time, and [6] proposes a hybrid intrusion detection system combining feature engineering with deep learning models.

However, a persistent challenge in the practical deployment of these techniques is the inherent class imbalance in network traffic data—especially in early-stage attack scenarios where benign traffic dominates and attack events are rare but critical to detect. This skewed distribution adversely affects model performance, as classifiers tend to be biased toward the majority class, resulting in poor sensitivity for detecting rare but significant attacks. Consequently, improving classification performance under class imbalance conditions has become a key research focus in sensor network security and anomaly detection applications—particularly in industrial control systems (ICSs). In such environments, stealthy or low-rate attacks may constitute only a tiny fraction of the traffic yet can cause significant operational disruptions if not promptly detected. Ref. [7] underscores the operational risks posed by low-rate attacks in SCADA systems, while [8] provides a case study on data imbalance issues in power grid intrusion detection.

To address the class imbalance problem, researchers have proposed a wide range of solutions broadly categorized into three approaches: data-level, algorithm-level, and hybrid methods. Refs. [9,10,11] survey these three categories and highlight the importance of hybrid solutions in real-time environments. At the data level, sampling techniques are widely used to balance class distributions either by oversampling the minority class or undersampling the majority class. The Synthetic Minority Oversampling Technique (SMOTE) and its variant SMOTE-ENN (SMOTE combined with Edited Nearest Neighbors) are among the most commonly used oversampling methods, as they generate synthetic samples of minority classes to balance datasets [12]. Ref. [13] enhances SMOTE by considering feature space density, while [14] integrates SMOTE with Tomek links to further refine class boundaries. Refs. [15,16] demonstrate the improved performance of SMOTE variants in IoT anomaly detection tasks. Conversely, undersampling approaches reduce the size of the majority class by randomly or selectively removing samples, thereby addressing imbalance without increasing data size. Ref. [7] applies random undersampling to industrial traffic data, and [17] proposes a density-aware undersampling method that retains structural diversity in the majority class.

On the algorithmic level, various methods have been proposed to incorporate class distribution into the learning process. Ref. [18] introduces cost-sensitive learning to penalize the misclassification of minority instances more heavily. Ref. [19] presents a boosting-based technique adapted for imbalanced settings, and [20] demonstrates the effectiveness of ensemble techniques in improving recall on minority classes. Hybrid methods that combine both data-level and algorithm-level strategies aim to achieve more robust performance by leveraging the strengths of both paradigms. Ref. [21] proposes a hybrid resampling technique integrated with ensemble classifiers, and [22] develops a framework that adapts both data sampling and classifier cost structure simultaneously. For example, ensemble models trained on resampled datasets or methods combining SMOTE with cost-sensitive algorithms have shown promising results in mitigating the effects of imbalance.

Despite these advancements, learning from imbalanced data remains a persistent challenge in real-world applications. Many methods suffer from overfitting due to excessive oversampling, while aggressive undersampling may discard informative samples. Additionally, existing techniques often do not adapt to the specific characteristics of the dataset. In the context of DDoS detection, this issue is even more pronounced. Although in most datasets attack traffic constitutes a minority class, there are also scenarios where attack traffic becomes the majority due to sampling or collection strategies. Ref. [8] identifies such bias in power grid datasets, while [23] shows that sampling artifacts can flip class proportions in real-time network logs. Such fluctuation in class ratios across datasets calls for more adaptive techniques that can accommodate varying degrees of imbalance while preserving critical decision boundaries. This dynamic nature of class distributions requires more flexible learning frameworks capable of adapting to varying imbalance ratios and maintaining performance across diverse datasets.

To overcome these limitations, we propose a novel adaptive sampling strategy tailored for DDoS traffic classification in imbalanced sensor datasets. Our method initially augments the minority class up to a predefined threshold using synthetic data generation, followed by the random undersampling of the majority class. Our framework incorporates a geometric refinement of SMOTE, named Geometric-SMOTE, which restricts synthetic data generation to safe regions around minority instances, thereby avoiding noisy or overlapping samples. The proposed method is primarily a data-level approach, as it focuses on rebalancing the dataset using advanced oversampling (Geometric-SMOTE) and filtering (ENN) techniques. However, by integrating an ensemble-based undersampling strategy during training, it also incorporates elements of algorithm-level adaptation. Therefore, the overall framework can be classified as a lightweight hybrid approach that combines the strengths of both data-level and algorithm-level methods.

This combined method effectively leverages both oversampling and noise reduction techniques, achieving superior accuracy, recall, and F1-scores compared to baseline models and traditional oversampling methods such as SMOTE and SMOTE-ENN. Experimental results confirm the robustness of the approach, particularly in datasets with dynamic or extreme imbalance conditions.

The remainder of this paper is organized as follows. Section 2 reviews the relevant literature on imbalanced learning techniques and DDoS traffic classification. Section 3 presents the proposed adaptive sampling framework, describing its methodological components and ensemble integration. Section 4 outlines the experimental setup, including a description of the DDoS datasets, preprocessing steps, parameter configurations, and evaluation metrics. Section 5 reports and discusses the experimental results, emphasizing the performance improvements achieved by the proposed approach. Finally, Section 6 concludes the paper by summarizing the main findings and suggesting directions for future research.

## 2. Related Work

Learning from imbalanced data has been a longstanding challenge in machine learning, particularly in security-related applications where critical events are rare but consequential. One of the most widely adopted approaches to this issue is the Synthetic Minority Oversampling Technique (SMOTE), which generates new synthetic samples by interpolating between a minority class instance and its nearest neighbors [12]. While SMOTE has become a standard baseline, its assumption of linearity between data points often leads to synthetic samples being placed in noisy or overlapping regions, especially when the minority class is not well-separated from the majority. To address these limitations, several SMOTE variants have been proposed. Borderline-SMOTE [24] focuses on samples near the decision boundary, while ADASYN [25] adaptively targets regions of low density. LR-SMOTE [26], for instance, improves upon the traditional SMOTE by generating synthetic samples that lie closer to the minority class center using local region constraints, thereby reducing the risk of creating outliers and preserving data distribution. More recent developments include geometric and density-aware oversampling methods that refine where and how synthetic instances are generated, aiming to preserve class topology and avoid introducing ambiguous points [16].

However, even advanced oversampling techniques can lead to degraded model performance if synthetic data are placed in regions of class overlap or near outliers. To mitigate this, postprocessing techniques such as Edited Nearest Neighbor (ENN) have been employed to clean the oversampled dataset. ENN removes samples—real or synthetic—that disagree with the majority of their local neighbors, thereby sharpening decision boundaries and reducing noise [13]. Hybrid methods such as SMOTE-ENN combine oversampling and noise reduction in a single pipeline and have demonstrated improved robustness in classification tasks with high imbalance. These approaches have proven especially useful in applications where the cost of misclassifying minority instances is high, such as fraud detection and cybersecurity.

Ensemble learning offers another effective strategy for handling imbalanced data. Techniques such as bagging and boosting have been adapted to incorporate class-weighting or cost-sensitive adjustments, allowing models to focus more heavily on minority class instances [19]. Additionally, undersampling-based ensembles construct multiple balanced training subsets by selectively reducing the majority class, which are then used to train individual base learners. These models are aggregated via voting or averaging to improve generalization. While some frameworks employ model-level integration schemes to enhance performance, our approach focuses on the complementary combination of geometric oversampling, noise filtering, and ensemble diversity to ensure robustness.

Although many of these techniques have been applied to standard imbalanced learning problems, relatively few studies have explicitly integrated geometric oversampling and noise filtering within a unified pipeline tailored for cybersecurity. Existing work in DDoS detection often emphasizes deep learning or feature engineering but lacks adaptive mechanisms for handling extreme or fluctuating class imbalance—particularly in sensor-driven or IoT environments where benign traffic can overwhelm rare but dangerous attacks. Our work addresses this gap by combining geometric sampling and ensemble integration in a manner that is both model-agnostic and sensitive to the structural properties of the data.

Recent advances in network intrusion detection highlight the need for solutions that are both adaptive and suitable for real-time or resource-constrained environments such as IoT systems. Zhong et al. [27] present a comprehensive survey on the use of graph neural networks (GNNs) for intrusion detection systems (IDSs), noting their effectiveness in modeling structured, dynamic network data. Gao et al. [28] show that deep learning architectures such as LSTM and feedforward networks can be successfully deployed in SCADA environments for real-time anomaly detection. Gueriani et al. [29] explore the use of deep reinforcement learning (DRL) to build IDSs capable of continuously adapting to new attack patterns in IoT networks. Hodo et al. [30] compare shallow and deep learning approaches to IDS design, revealing trade-offs in complexity, detection speed, and interpretability. A recent study by Gelenbe et al. [31] proposes DISFIDA, a distributed, self-supervised federated learning framework for intrusion detection in health IoT and vehicular networks, emphasizing its capacity for online adaptation. Meanwhile, FN-GNN [32] introduces a graph embedding enhancement technique to improve the robustness of GNN-based IDSs under variable network topologies. Finally, recent applications of Dueling Double Deep Q-Learning in IDS architectures [33] show promising results in fast-evolving traffic scenarios, offering low-latency decisions without retraining. Collectively, these studies underline the increasing emphasis on real-time, adaptable detection methods that can operate under diverse and evolving network conditions. While our proposed framework is designed for batch-mode training, its modular design—particularly the decoupling of oversampling and filtering components—allows potential adaptation to online learning pipelines.

## 3. Proposed Method

We introduce an effective algorithm for detecting DDoS attacks, called the *adaptive sampling method*, which is explained through three pseudocodes in the following subsections.

### 3.1. Synthetic Data Generation via Geometric-SMOTE

To address the severe class imbalance between benign and attack traffic, we developed a refined oversampling strategy called **Geometric-SMOTE**, inspired by the well-known SMOTE. These oversampling methods aim to artificially increase the number of minority class (attack) instances without simply duplicating existing data, thereby enhancing model generalization.

SMOTE is a widely used data-level approach to mitigate class imbalance in classification problems. Instead of replicating existing minority samples, SMOTE generates synthetic instances by interpolating between a sample and one of its nearest minority class neighbors. Given a minority instance, *x*, a neighbor, $x_{\mathrm{nn}}$, is randomly selected, and a new instance is created as follows:

$$x_{\mathrm{new}}=x+\lambda \cdot \left(x_{\mathrm{nn}}-x\right),\;\lambda \in \left[0,1\right].$$

This technique introduces diversity by creating synthetic samples along line segments between neighboring points in the feature space. As a result, it avoids overfitting commonly associated with naive oversampling strategies and helps the model generalize better.

Over time, various enhanced versions of SMOTE have been proposed, such as Borderline-SMOTE [24], LR-SMOTE [26], and MeanRadius-SMOTE [16]. These variants focus on generating samples near decision boundaries or use more informed geometric criteria to avoid the creation of noisy or less informative samples. While these improvements reduce some of SMOTE’s limitations, challenges remain—particularly in generating synthetic points near outliers or in overlapping regions with the majority class, where noise and ambiguity may be introduced.

To overcome these limitations, we propose **Geometric-SMOTE**, a geometric extension of SMOTE that generates synthetic samples within locally adaptive, safe regions around minority instances, as detailed in Algorithm 1. Specifically, for each minority instance *x*, we compute the distance to its nearest majority class sample:

(1) $$r_{x}=\min_{x^{\prime }\in M}∥x-x^{\prime }∥$$

where M denotes the set of majority class instances and ∥·∥ represents the Euclidean norm. This distance defines the radius of an open ball, $B(x,r_{x})$, centered at *x*:

$$B\left(x,r_{x}\right)=\{y\in R^{d}:\;∥y-x∥\;<\;r_{x}\}.$$

**Figure 1:**
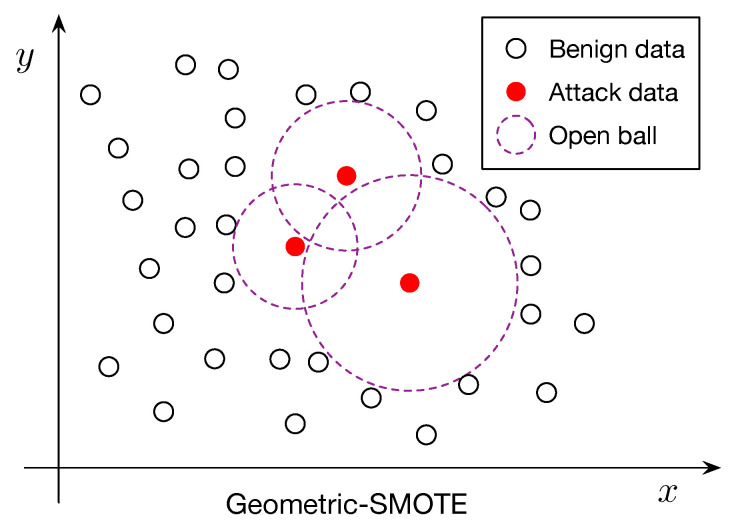
Geometric-SMOTE diagram for the construction of synthetic samples.

Synthetic samples are then generated at random within the union of such open balls:

(2) $$B=\underset{x\in S}{⋃}B\left(x,r_{x}\right)$$

where S is the set of minority class instances selected for oversampling (see Figure 1 for an illustration).

The minority class is augmented by a factor of α. That is, the augmentation continues until the ratio α, defined by

(3) $$\alpha =\frac{|S_{\mathrm{new}}|}{\left|S\right|}$$

reaches a target proportion, where |S| represents the size of the original minority class samples and $|S_{\mathrm{new}}|$ is the size of the both original and synthetic samples of the minority class. For example, α=2 generates the same number of synthetic samples as the number of original minority samples so that the total number of minority samples becomes doubled.

Compared to Borderline-SMOTE and SMOTE-Tomek, Geometric-SMOTE provides more controlled synthetic data generation by using locally defined safe regions. This helps avoid noise and preserves the geometric structure of the minority class. The addition of ENN filtering further enhances sample quality, differentiating our method from conventional hybrid techniques. Table 1 summarizes the differences.

By restricting generation to these adaptive, majority-free regions, Geometric-SMOTE reduces the risk of introducing ambiguous or borderline samples. Unlike SMOTE, where generated samples lie along straight lines between observed instances, Geometric-SMOTE introduces nonlinear variability by sampling from regions defined relative to the surrounding majority class distribution. Consequently, synthetic instances from Geometric-SMOTE can be viewed as a nonlinear mapping of the original data, offering richer representation and better approximating the underlying distribution of the minority class.
**Algorithm 1:** Geometric-SMOTE
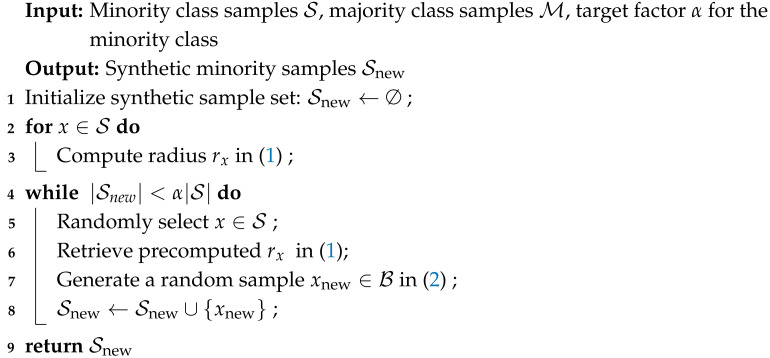


### 3.2. ENN-Based Filtering

After generating synthetic attack samples, we apply a filtering step to reduce the risk of introducing mislabeled or ambiguous data points. Specifically, we utilize the Edited Nearest Neighbor (ENN) technique, as detailed in Algorithm 2, to eliminate synthetic samples that are likely to resemble benign data.

In this filtering process, for each synthetic attack sample, we identify its *k* nearest neighbors from the combined dataset of real benign and attack data. If the majority of these *k* neighbors are benign instances, we consider the synthetic point to be potentially misrepresentative of the attack class and remove it from the training set.

This strategy effectively reduces the overlap between synthetic attack data and benign data in feature space, thereby enhancing the discriminative quality of the training dataset. The ENN-based filtering also helps suppress the false positive rate by removing noisy synthetic samples that could confuse the classifier during training.
**Algorithm 2:** ENN-based filtering
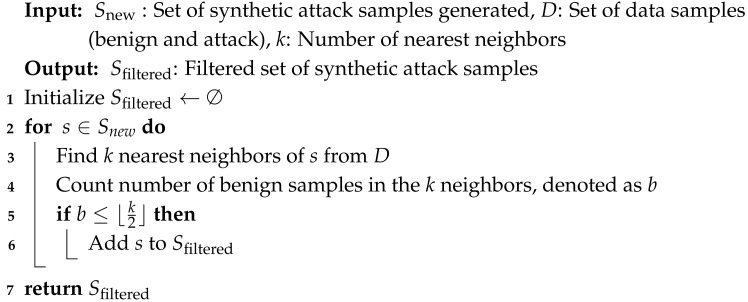


### 3.3. Undersampling Ensemble Strategy

To further mitigate the impact of class imbalance during training, we employ an undersampling ensemble strategy that constructs multiple balanced training datasets by repeatedly selecting different subsets of the majority class, as detailed in Algorithm 3. This technique complements our oversampling approach and enhances model robustness. Unlike traditional undersampling techniques that randomly discard majority class instances, our method adopts an ensemble-based undersampling strategy. This approach constructs multiple balanced training sets by sampling diverse subsets of the majority class, thereby enhancing generalization and reducing the risk of losing informative samples. Combined with Geometric-SMOTE and ENN filtering, the overall framework effectively integrates both oversampling and undersampling innovations within a unified data-level pipeline.

The procedure operates in three main stages. First, after applying oversampling techniques (e.g., Geometric-SMOTE in Algorithm 1 and ENN in in Algorithm 2) to augment the minority class instances, we randomly select β% of the majority class (benign samples). These two subsets are combined to form a balanced training set. Second, we train a classifier (e.g., Random Forest) on this balanced training data and evaluate the resulting model on the test set. Third, the first two steps are repeated multiple times, each time selecting a different random subset of majority class data. Finally, the outputs of all classifiers are aggregated using majority voting to produce the final prediction for each test instance.

This ensemble-based undersampling strategy provides several benefits over naive random undersampling. First, by constructing multiple balanced training sets with different majority subsets, the method reduces the risk of information loss that may occur when discarding majority samples in a single-shot manner. Second, this process increases diversity among base classifiers, which improves the generalization capability of the ensemble. Third, the majority voting aggregation reduces variance and overfitting to specific undersampled configurations. As a result, the classifier can capture more robust decision boundaries, especially in highly imbalanced and noisy settings. This approach is model-agnostic and can be easily integrated with various base learners.
**Algorithm 3:** Undersampling ensemble strategy
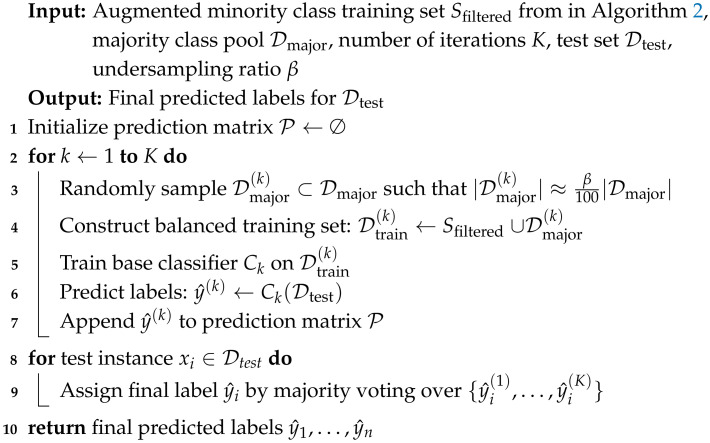


**Figure 2:**
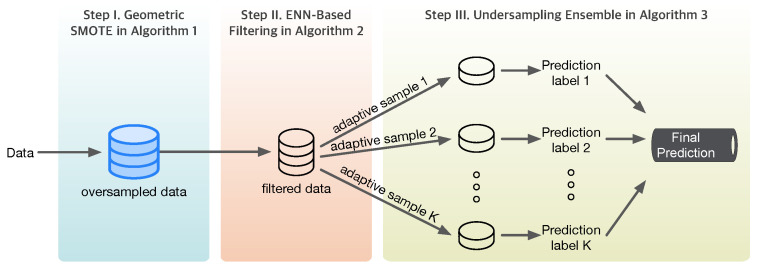
Proposed adaptive sampling algorithm.

The flowchart of the adaptive sampling algorithm, summarized from the contents of Algorithms 1–3, is shown in the following Figure 2.

## 4. Experimental Setup

### 4.1. Dataset Construction

To evaluate the proposed classification framework, we constructed datasets by combining benign and DDoS attack traffic obtained from sensor-based network environments, downloaded from Kaggle [34]. Four attack types are considered in this study: NetBIOS, SYN, UDP, and UDPLag. Details regarding these attacks can be found on the Kaggle platform. Table 2 summarizes the features in the raw data.

Given the inherent class imbalance—where attack samples constitute less than 5% of the total instances—we adopted a systematic process to prepare the dataset for model training and evaluation. To simulate realistic deployment scenarios, we randomly selected a limited subset of attack samples from the full dataset. This approach allowed us to focus on effective detection strategies under highly imbalanced conditions, reflecting real-world environments where the timely identification of rare attack traffic is critical. The ratio of attack to benign data is summarized in Table 3.

### 4.2. Machine Learning Models and Training Configuration

This study employed two machine learning models selected for their effectiveness in handling structured data and class imbalance: Random Forest (RF) and Extreme Gradient Boosting (XGBoost). Each model was independently trained on balanced datasets created through the oversampling and undersampling techniques described earlier.

*Random Forest* is an ensemble learning algorithm that constructs multiple decision trees using bootstrapped subsets of the training data. We used 100 trees, each with a maximum depth of 10, and Gini impurity as the splitting criterion.

*Extreme Gradient Boosting (XGBoost)* is a regularized gradient boosting framework optimized for speed and performance. It is configured with 200 boosting rounds, a learning rate of 0.1, and a maximum tree depth of 6. Early stopping with a patience of 10 rounds was employed to prevent overfitting.

All models were implemented using Python with Scikit-learn 1.7.0.

### 4.3. Evaluation Metrics

To comprehensively evaluate the performance of our classification framework, we utilize a set of widely adopted evaluation metrics for binary classification. These include *accuracy*, *precision*, *recall*, and *F1-score*. Each metric offers a unique perspective on classifier performance, especially under imbalanced conditions, which are typical in DDoS detection scenarios.

*Accuracy* measures the overall proportion of correctly predicted instances:

$$\mathrm{Accuracy}=\frac{\mathrm{TP}+\mathrm{TN}}{\mathrm{TP}+\mathrm{FP}+\mathrm{TN}+\mathrm{FN}}$$

Although easy to interpret, accuracy can be misleading in highly imbalanced datasets, as it may be dominated by the majority class.

*Precision* quantifies the proportion of predicted positive instances that are actually positive:

$$\mathrm{Precision}=\frac{\mathrm{TP}}{\mathrm{TP}+\mathrm{FP}}$$

High precision indicates a low false positive rate, which is critical in minimizing incorrect attack detections.

*Recall*, or *sensitivity*, is the proportion of actual positive instances that are correctly identified:

$$\mathrm{Recall}=\frac{\mathrm{TP}}{\mathrm{TP}+\mathrm{FN}}$$

In the context of DDoS detection, high recall is especially important, as failing to detect attack traffic (false negatives) can lead to severe consequences.

The *F1-score* is the harmonic mean of precision and recall:

$$F1-\mathrm{score}=2\cdot \frac{\mathrm{Precision}\cdot \mathrm{Recall}}{\mathrm{Precision}+\mathrm{Recall}}$$

It provides a balanced measure when both false positives and false negatives are important, which is often the case in cybersecurity applications.

In summary, while all metrics are reported for completeness, we emphasize recall and F1-score due to their practical relevance in imbalanced attack detection settings.

## 5. Experimental Results

We compare the detection performance of four data sampling configurations:

1. Raw (no sampling);
2. SMOTE + ENN (oversampling only);
3. SMOTE + ENN + undersampling (combined sampling);
4. Geometric-SMOTE + ENN + undersampling (adaptive sampling).

Each method was evaluated using four metrics and two machine learning algorithms with four attack types, as detailed in Table 4. Oversampling with SMOTE and Geometric-SMOTE constructed synthetic samples to increase the number of minority samples by a factor of α. Undersampling selected β% of the majority class at random, where β= None implied that undersampling was not performed and only oversampling was performed. The experiments with undersampling were repeated three times (K=3) and the final results are reported as the average performance across these runs.

Table 4 summarizes the parameters used.

Table 5 and Table 6 present detailed classification performance across different attack types and sampling strategies for Random Forest and XGBoost classifiers, respectively. Across both models, we observe that **adaptive sampling**—which combines Geometric-SMOTE with ENN filtering and undersampling—consistently yielded the best overall performance, particularly in recall and F1-score.

For well-represented attacks such as *UDP*, both classifiers performed well even without sampling, achieving high accuracy, precision, and recall. However, for under-represented and difficult cases like *Syn* and *UDPLag*, models trained on raw or oversampled data failed to detect attacks effectively, as evidenced by recall scores near zero. In contrast, adaptive sampling drastically improved detection: Random Forest achieved an F1-score of 0.994 for *Syn* and 0.948 for *UDPLag*, while XGBoost reached perfect detection with an F1-score of 1.000 for *Syn* and 0.997 for *UDPLag*.

Between the two classifiers, XGBoost generally showed superior performance on imbalanced or borderline cases. For instance, in the *NetBIOS* and *UDPLag* attacks under adaptive sampling, XGBoost yielded higher F1-scores than Random Forest.

In summary, these results confirm that (1) imbalanced data leads to severe underperformance for rare attacks, (2) traditional oversampling alone is insufficient for robust detection, and (3) adaptive sampling methods offer significant benefits by enabling models to better capture minority class characteristics.

**Figure 3:**
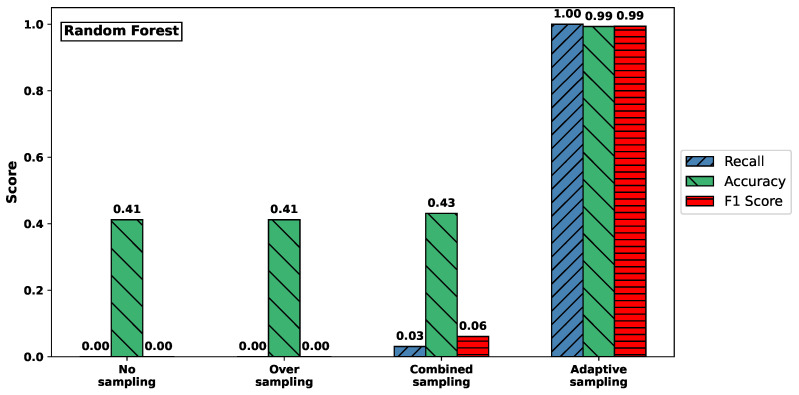

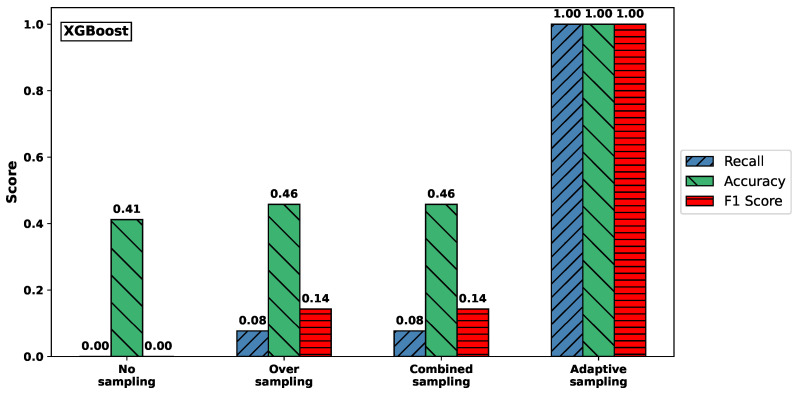
Comparison of recall, accuracy, and F1-score for Syn attack across sampling methods. (**Top**) RF. (**Bottom**) XGBoost.

Figure 3 visualizes the effect of different sampling methods on the classification performance for the *Syn* attack type, using Random Forest (top) and XGBoost (bottom) classifiers. The plots report recall, accuracy, and F1-score, highlighting how these metrics vary with respect to sampling strategies.

For both classifiers, models trained on the original imbalanced dataset or using only oversampling failed to detect any *Syn* attacks, resulting in recall values close to zero. In contrast, adaptive sampling substantially improved recall: for Random Forest, it rose from 0.000 to 1.000; and for XGBoost, from 0.077 to 1.000. At the same time, accuracy also increased dramatically, from approximately 0.41–0.46 to nearly perfect classification performance (0.993 and 1.000, respectively).

These results clearly demonstrate that adaptive sampling is crucial for enabling both classifiers to detect rare attack types like *Syn*, which would otherwise be completely overlooked under naive training conditions.

**Figure 4:**
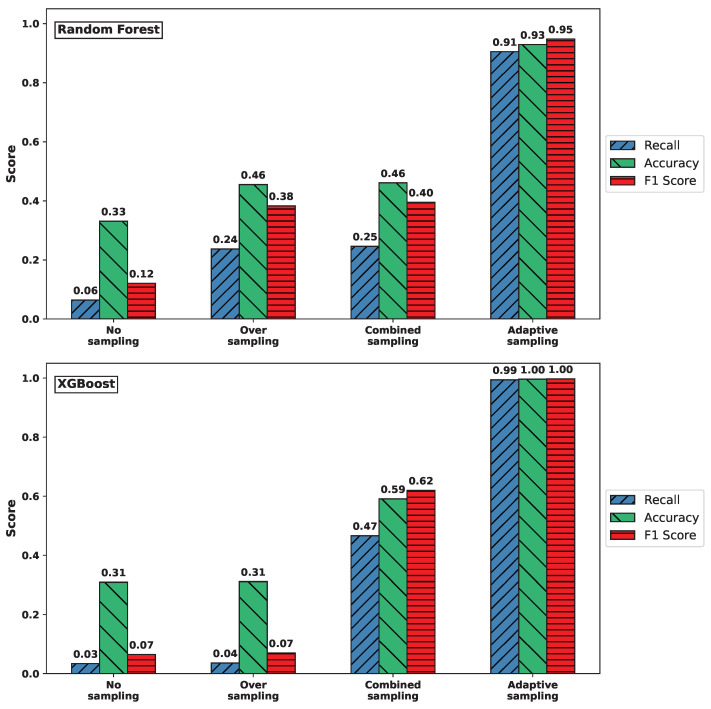
Comparison of recall, accuracy, and F1-score for UDPLag attack across sampling methods. (**Top**) RF. (**Bottom**) XGBoost.

Figure 4 shows that similar performance patterns were observed for the UDPlag attack data. In particular, the adaptive sampling method yielded improved recall, accuracy, and F1-score values compared to the other sampling strategies.

**Figure 5:**
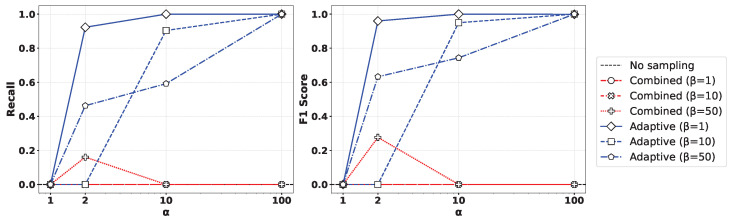
Comparison of (**left**) recall and (**right**) F1-score for various values of the oversampling ratio α and undersampling parameter β% by applying the XGBoost method to the Syn attack data.

**Figure 6:**
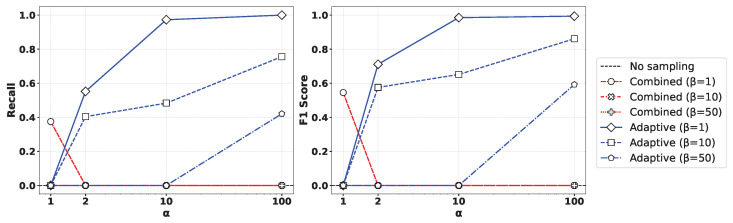
Comparison of (**left**) recall and (**right**) F1-score for various values of the oversampling ratio α and undersampling parameter β% by applying the Random Forest method to the Syn attack data.

Figure 5 presents the performance comparison of (left) recall and (right) F1-score for various values of the oversampling ratio α and undersampling parameter β% when applying the XGBoost algorithm to the Syn attack data. As observed in the figure, the Geometric-SMOTE-based oversampling approach showed a clear trend where both recall and F1-score converged toward 1 as α increased. In contrast, other oversampling methods did not achieve comparable performance, even with high oversampling ratios. Moreover, lower values of β led to more balanced training datasets, which in turn yielded improved classification results. Similar results can be observed in Figure 6, presenting the outcome of applying the Random Forest method.

## 6. Conclusions

This study addresses the challenge of detecting DDoS attacks in highly imbalanced sensor network traffic by evaluating the effectiveness of various sampling strategies within a modular classification framework. We compared four approaches: using the raw imbalanced dataset, applying SMOTE + ENN for oversampling, applying SMOTE + ENN with undersampling, and applying Geometric-SMOTE with adaptive undersampling.

Experimental results show that models trained on raw data suffered from poor recall values. The application of SMOTE + ENN improved recall marginally. The adaptive sampling strategy that combines Geometric-SMOTE and random undersampling consistently achieved the best balance between precision and recall, resulting in the highest F1-score.

While the current evaluation was offline, all major sampling operations were performed during training. Thus, the deployed model remains lightweight and suitable for online inference. Future extensions will explore integration with streaming frameworks for real-time anomaly detection in sensor-driven environments.

## Figures and Tables

**Table 1:** Comparison of oversampling techniques.

Method	Key Idea	Advantages	Limitations	Comparison to Geometric-SMOTE
SMOTE	Linear interpolation between minority neighbors	Simple and widely used	May generate samples in noisy or overlapping regions	Geometric-SMOTE restricts generation to safe geometric regions
Borderline -SMOTE	Focuses on samples near decision boundary	Improves focus on difficult samples	Vulnerable to noise and poorly defined boundaries	Geometric-SMOTE avoids noisy boundaries via geometric constraints
SMOTE -Tomek	SMOTE and removal of Tomek links (overlapping instances)	Reduces class overlap	May remove informative borderline samples	Geometric-SMOTE retains more informative samples by filtering only unsafe regions
SMOTE -ENN	SMOTE + ENN to remove noisy samples	Enhances quality of synthetic data	Filtering may be too aggressive in sparse regions	Geometric-SMOTE applies ENN after geometrically safe sampling, reducing noise
Geometric -SMOTE + ENN	Samples generated in locally defined safe regions and ENN filtering	Preserves class topology; avoids noise; adapts to local structures	Slightly higher computational cost due to distance calculations	Combines strengths of geometry-based control and noise filtering

**Table 2:** Features in the raw data.

Class	Features
Categorical	Protocol, Fwd PSH Flags, Fwd URG Flags, FIN Flag Count,
	SYN Flag Count, etc.
Numerical	Flow Duration, Total Fwd Packets, Fwd Packets Length Total,
	Flow Bytes/s, Flow Packets/s, etc.

**Table 3:** The ratio of attack to benign data.

Class	NetBIOS	SYN	UDP	UDPLag
Ratio (%)	0.2433	0.2996	0.2824	0.2935

**Table 4:** Parameters used in the experiments.

Parameter	Values
metrics	accuracy, precision, recall, F1-score
machine learning algorithms	Random Forest, XGBoost
attack types	NetBIOS, Syn, UDP, UDPLag
α (oversampling)	1, 2, 10, 100
β% (undersampling)	None, 1, 10, 50

**Table 5:** Performance metrics of Random Forest for each attack type and sampling method.

Attack	Method	Accuracy	Precision	Recall	F1-Score
NetBIOS	No sampling	0.851	**1.000**	0.446	0.617
	Oversampling	0.882	**1.000**	0.561	0.719
	Combined sampling	0.991	0.978	0.989	0.983
	Adaptive sampling	**0.994**	0.987	**0.991**	**0.989**
Syn	No sampling	0.412	0.000	0.000	0.000
	Oversampling	0.412	0.000	0.000	0.000
	Combined sampling	0.431	**1.000**	0.031	0.061
	Adaptive sampling	**0.993**	0.988	**1.000**	**0.994**
UDP	No sampling	0.979	**1.000**	0.975	0.987
	Oversampling	0.987	**1.000**	0.984	0.992
	Combined sampling	0.998	0.999	**0.999**	**0.999**
	Adaptive sampling	**0.999**	**1.000**	**0.999**	**0.999**
UDPLag	No sampling	0.331	**1.000**	0.064	0.121
	Oversampling	0.455	**1.000**	0.237	0.383
	Combined sampling	0.461	**1.000**	0.246	0.395
	Adaptive sampling	**0.929**	0.995	**0.905**	**0.948**

**Table 6:** Performance metrics of XGBoost for each attack type and sampling method.

Attack	Method	Accuracy	Precision	Recall	F1-Score
NetBIOS	No sampling	0.974	**1.000**	0.903	0.949
	Oversampling	0.974	**1.000**	0.903	0.949
	Combined sampling	0.982	0.986	0.944	0.965
	Adaptive sampling	**0.995**	0.994	**0.987**	**0.991**
Syn	No sampling	0.412	0.000	0.000	0.000
	Oversampling	0.458	**1.000**	0.077	0.143
	Combined sampling	0.458	**1.000**	0.077	0.143
	Adaptive sampling	**1.000**	**1.000**	**1.000**	**1.000**
UDP	No sampling	0.988	**1.000**	0.985	0.993
	Oversampling	0.988	**1.000**	0.985	0.993
	Combined sampling	0.988	**1.000**	0.985	0.993
	Adaptive sampling	**0.999**	**1.000**	**0.999**	**0.999**
UDPLag	No sampling	0.309	**1.000**	0.034	0.065
	Oversampling	0.311	**1.000**	0.036	0.070
	Combined sampling	0.591	0.925	0.466	0.620
	Adaptive sampling	**0.996**	**1.000**	**0.994**	**0.997**

## Data Availability

The dataset is available on request from the authors.

## Abbreviations

The following abbreviations are used in this manuscript:

DDoS	Distributed Denial of Service
SMOTE	Synthetic Minority Oversampling Technique
SMOTE-ENN	SMOTE combined with Edited Nearest Neighbor
RF	Random Forest
XGBoost	Extreme Gradient Boosting
